# Hyperoxaemia and hypoxaemia are associated with harm in patients with ARDS

**DOI:** 10.1186/s12890-021-01648-7

**Published:** 2021-09-08

**Authors:** Andrew J. Boyle, David N. Holmes, Jonathan Hackett, Susanna Gilliland, Michael McCloskey, Cecilia M. O’Kane, Paul Young, Stefania Di Gangi, Daniel F. McAuley

**Affiliations:** 1 Regional Intensive Care Unit, Royal Victoria Hospital, Belfast, Northern Ireland; 2 Centre for Experimental Medicine, Queen’s University Belfast, 97 Lisburn Road, Belfast, Northern Ireland; 3 Department of Intensive Care Medicine, Wellington Regional Hospital, Wellington, New Zealand; 4 Medical Research Institute of New Zealand, Wellington, New Zealand; 5 Institute of Primary Care, University Hospital Zürich, Pestalozzistrasse 24, 8091 Zürich, Switzerland

**Keywords:** Acute respiratory distress syndrome, Hyperoxia, Hyperoxaemia, Oxygen, Excess oxygen

## Abstract

**Background:**

Oxygen therapy is routinely administered to mechanically ventilated patients. However, there remains uncertainty about the optimal oxygen titration target in patients with the acute respiratory distress syndrome (ARDS).

**Methods:**

Prospectively identified adult patients meeting the Berlin definition of ARDS between 1st January 2014 and 13th December 2016 were analyzed. Oxygen exposure variables were collected at 6-hourly intervals. The primary exposure was the average time-weighted partial pressure of arterial oxygen (PaO_2_) calculated over a maximum of 7 days from meeting ARDS criteria. The primary outcome was ICU mortality. Univariable and multivariable logistic regression analyses were performed to assess the impact of exposure variables on clinical outcomes. Results are presented as odds ratio [95% confidence interval].

**Results:**

202 patients were included in the final analysis. Overall ICU mortality was 31%. The average time-weighted PaO_2_ during the first 7 days of ARDS was similar between non-survivors and survivors (11.3 kPa [10.2, 12.5] (84.8 mmHg [76.5, 93.8]) vs. 11.9 kPa [10.9, 12.6] (89.3 mmHg [81.8, 94.5]); *p* = 0.08). In univariable and multivariable analysis, average time-weighted PaO_2_ demonstrated a U-shaped relationship with ICU mortality. There was a similar relationship identified with hospital mortality.

**Conclusions:**

In patients with ARDS, the predicted probability of both ICU and hospital mortality was lowest when the average time-weighted PaO_2_ was between 12.5 and 14 kPa (93.8–105.0 mmHg), suggesting this is a reasonable oxygenation target for clinicians to aim for.

**Supplementary Information:**

The online version contains supplementary material available at 10.1186/s12890-021-01648-7.

## Background

Supplemental oxygen therapy is routinely required as part of the supportive care of patients with the acute respiratory distress syndrome (ARDS), but it is typically administered without defined or standardized targets [1]. This has prompted oxygenation targets in ARDS to be considered a priority for clinical trials in ARDS [2].

The mechanisms through which oxygen therapy may be injurious are unclear, and it is difficult to separate the potentially injurious effects of high fractions of inspired oxygen (FiO_2_) causing alveolar hyperoxia, from high partial pressure of arterial oxygen (PaO_2_) causing hyperoxaemia. Exposure to supra-physiological FiO_2_ (0.5–0.6) has been demonstrated to augment lipopolysaccharide and ventilator-induced lung injury in pre-clinical models [3, 4]. The potentially harmful effects of alveolar hyperoxia were also demonstrated in a clinical trial of mechanically ventilated patients with septic shock. Although this trial was discontinued early, there were fewer ventilator-free days and more serious adverse events in the group allocated to receive maximal oxygen therapy (FiO_2_ 1.0) [5].

Hyperoxaemia has also been associated with harm in critically ill patients in multiple studies [6–10]. In an open-label randomized controlled trial of adult patients admitted to the intensive care unit (ICU), conservative oxygen therapy (defined as the lowest possible FiO_2_ to maintain PaO_2_ between 9.3 and 13.3 kPa (70–100 mmHg) or arterial oxyhemoglobin saturation measured by pulse oximetry (SpO_2_) 94–98%) was reported to reduce ICU mortality when compared with standard care [11]. Furthermore, in a cohort of patients receiving veno-venous extracorporeal membrane oxygenation for respiratory failure, moderate hyperoxaemia (PaO_2_ between 13.5 and 40 kPa (101–300 mmHg)) was associated with increased hospital mortality [12].

However, in a large randomized controlled trial of mechanically ventilated adults, a conservative oxygen strategy titrated to avoid hyperoxaemia did not reduce ventilator-free days [13]. Whilst this suggests that conservative oxygen targets may be as safe as usual care, it does not exclude the possibility of harm from hyperoxaemia. Similarly, in patients with acute hypoxaemic respiratory failure who were admitted to the ICU, which included some patients with ARDS, there was no difference in outcome between patients allocated to receive an oxygen target of either 8 kPa (60 mmHg) or 12 kPa (90 mmHg) [14]. In comparison, a clinical trial of patients with early ARDS, allocation to conservative oxygen therapy (PaO_2_ target 7.3–9.3 kPa (55–70 mmHg)) was associated with higher 90-day mortality than a liberal oxygen strategy (PaO_2_ target 12–14 kPa (90–105 mmHg)) [15]. Despite these results, a post-hoc analysis of 10 randomized controlled trials that recruited patients with ARDS provides conflicting data, suggesting that higher cumulative excess oxygen exposure (sum of inspired oxygen received beyond what was required for a specified PaO_2_) was associated with increased hospital mortality and fewer ventilator-free days [16].

These data demonstrate that there is still uncertainty about the optimal oxygen titration targets for patients with ARDS. Whilst most studies have investigated a strategy of conservative or liberal oxygen titration strategies, it is plausible that there is an optimal target for oxygenation between the ranges studied thus far. In an evaluation of prospectively identified patients, the primary aim of this analysis was to assess whether there was a relationship between average time-weighted PaO_2_ and ICU mortality in patients with ARDS. Secondary aims included evaluating whether oxygenation targets were associated with changes in hospital mortality or duration of invasive mechanical ventilation.

## Methods

### Patient population

An observational study of prospectively identified adult patients (> 16 years, with no upper age limit) requiring invasive mechanical ventilation admitted to a 27-bed mixed medical and surgical intensive care unit was performed. Between 1st January 2014 and 13th December 2016, patients were prospectively evaluated each day during their admission. All patients meeting the Berlin definition of ARDS [17], regardless of severity, with at least 12 h of available data were included in the study. This was a convenience sample, and the timeframe selected represented the onset of patient identification and planning for ARDS clinical trials. Data collection ceased when staff were no longer available to work on this project. As such, a power calculation was not performed. This study was conducted within the Belfast Health and Social Care Trust, who determined this study as an audit because patient management was not altered, only routinely collected data were used and the data were fully anonymized. As a result, research ethics committee approval was not required.

### Data collection

Baseline demographics recorded at the time of ICU admission included age, sex, source of admission (e.g. elective, emergency, medical or surgical) and acute physiology and chronic health evaluation (APACHE) II score. Non-respiratory sequential organ failure assessment (SOFA) scores were calculated at the time of ARDS diagnosis. Predicted body weight (PBW) was calculated for each patient based on their documented height and sex [18], and from this baseline tidal volume per predicted body weight (ml/kg) was determined. Additional parameters recorded at diagnosis of ARDS included blood lactate, positive end expiratory pressure (PEEP), fraction of inspired oxygen (FiO_2_), partial pressure of arterial oxygen (PaO_2_), arterial oxygen saturation (SaO_2_) and mean airway pressure (MAP). P/F ratio was calculated from available arterial blood gas measurements at 6-hourly intervals. Oxygenation values are expressed in kPa (mmHg) throughout the manuscript, with the equivalent value in mmHg accompanying kPa values in each results table.

### Exposures

Oxygen exposure variables were collected from the onset of ARDS for 7 days, or until unassisted breathing (defined as extubation, or breathing without ventilatory assistance with a PEEP ≤ 5 cm H_2_O for one whole calendar day) or death (whichever occurred earliest). These variables were collected for up to 7 days because early oxygen exposure has previously been demonstrated to have significant effect on outcomes in other cohorts of critically ill patients [5, 11, 19]. The primary exposure of interest was the average time-weighted PaO_2_ calculated over a maximum of 7 days from meeting ARDS criteria [20]:

$${\text{Average}}\;{\text{time-weighted}}\;{\text{PaO}}_{{2}} \frac{{{\text{T-PaO}}_{{2}} {1} + {\text{T-PaO}}_{{2}} {2} + \cdots {\text{T - PaO}}_{{2}} x}}{{{\text{T-Total}}}}$$

T-PaO_2_ = mean PaO_2_ × time (between consecutive timepoints where PaO_2_ was measured). T-Total = time between the first and last PaO_2_ measurement during the period of mechanical ventilation after meeting ARDS criteria, up to 7 days.

Hyperoxaemia was defined prospectively as a PaO_2_ greater than 14 kPa (105 mmHg) [11]. Excess oxygen exposure was defined as any FiO_2_ > 0.5 in patients with a PaO_2_ > 10.7 kPa (80 mmHg), and was calculated at 24-h intervals with a cumulative value obtained [16].

### Outcomes

The primary outcome was ICU mortality. Secondary outcomes included duration of ventilation and hospital mortality (both censored at 60 days). Duration of ventilation was calculated as the total time of invasive mechanical ventilation until unassisted breathing was achieved for one whole calendar day [21].

### Statistical methods

Baseline characteristics and outcome variables were compared using standard tests for continuous and binary variables. Data are presented as means (standard deviation, SD), median (interquartile range, [IQR]) and number (percentage, %) as appropriate. The percentage of missing observations is shown when necessary. The distributions of all variables were tested for normality. The independent sample Student’s t-test was used for continuous variables with a normal distribution. Otherwise, the Wilcoxon‐Mann‐Whitney was used when normality was violated. The Chi squared test or Fisher’s exact two-sided test, as appropriate, were used for binary variables. A *p* < 0.05 was considered statistically significant.

Univariable and multivariable logistic regression models were performed to identify the association between baseline characteristics and, respectively, ICU mortality, hospital mortality, and duration of mechanical ventilation. Results of regression models for ICU and hospital mortality are shown as odds ratio (OR) [95% confidence interval (CI)]. Duration of mechanical ventilation is presented in log scale. The relationship between mortality and either average time-weighted PaO_2_ or highest PaO_2_ was modelled through a quadratic trend. Results of the models with duration of mechanical ventilation as outcomes are shown as Estimate [95% CI]. Values of the estimate are values of the estimated beta coefficient of predictors, and therefore positive coefficient values mean that for a unit increase in the predictor, the outcome is increasing, and for negative coefficient values the outcome is decreasing. Multivariable models were constructed using variables that showed a statistically significant difference (*p* < 0.2) between the groups in univariate analysis. Stepwise backward elimination was used to develop final multivariable models with best fit for the outcome of interest. As the primary exposure of interest, average time-weighted PaO_2_ within the first 7 days of ARDS was forced into multivariable models evaluating ICU and hospital mortality. Where variables were subject to collinearity, a preference was made to select average-time weighted PaO_2_ in the models because it was the primary exposure of interest.

Mediation analysis [22] was performed to identify if there was a causal relationship between an early exposure (hyperoxaemia in the first 24 h from ARDS diagnosis), high lactate or baseline tidal volume (mediators), and how this relationship affected ICU and hospital mortality. Early hyperoxaemia was selected as an exposure because it was an potential early event in the disease course that has been previously demonstrated to be associated with increased mortality in mechanically ventilated patients without ARDS [19]. High lactate was chosen as a mediator because prior analyses have identified that there is an increased risk of death in patients with septic shock and a high lactate exposed to hyperoxia, whilst baseline tidal volume was selected because volutrauma has been shown to augment experimental hyperoxia-induced lung injury [4]. Baseline FiO_2_ and PEEP were considered as confounders based on their clinical effect on oxygenation. The causal relationship was assessed in three steps: (1) indirect or mediated effect: regression models with the two mediators (high lactate and baseline tidal volume) as dependent variables and the exposure (hyperoxaemia in the first 24 h) as the independent variable together with baseline FiO_2_ and PEEP as confounders; (2) effect of mediators on outcomes: regression model with ICU or hospital mortality as dependent variable and each mediator and confounders as independent variables; (3) estimation of mediators effect as a combination of both steps 1 and 2. All statistical analyses were carried out using the R statistical package (https://www.R-project.org).

## Results

Between 1st January 2014 and 13th December 2016, 3773 patients were admitted to the ICU, and of these, 222 patients were identified for inclusion. Following clinical review for suitability, 202 patients were confirmed as having ARDS with at least 12 h of available data, and therefore included in the final analysis. A full list of exclusions is provided in the Additional file 1. The median duration of mechanical ventilation in patients was 9 days [5, 16]. Overall ICU mortality was 31%, and overall hospital mortality was 38%. At the point of meeting ARDS criteria, ICU survivors were younger, had a lower non-respiratory SOFA score, and had a lower blood lactate than non-survivors (Table 1).

**Table 1:** Baseline characteristics

Variables	Overall (N = 202)	Non-Survivors (N = 63; 31%)	Survivors (N = 139; 69%)	*p*-value
Age (years)	59.0 [43.0, 71.0]	69.00 [52.0, 76.5]	56.0 [39.0, 67.0]	< 0.001
Male—N (%)	127 (62.9)	38 (60)	89 (64)	0.73
APACHE II^a^	19.0 [15.0, 23.0]	21.0 [17.0, 24.0]	18.0 [15.0, 23.0]	0.15
Non-respiratory SOFA score	8.00 [5.00, 10.0]	8.00 [7.0, 11.0]	7.0 [4.0, 9.5]	0.03
*Admission source*–*N (%)*				
Emergency	145 (71.8)	46 (73)	99 (71)	0.92
Elective	11 (5.4)	3 (4.8)	8 (6)	1.00
Medical	89 (44.1)	27 (43)	62 (45)	0.94
Surgical	71 (35.1)	26 (41)	45 (32)	0.29
*ARDS severity*—*N (%)*				
Mild	78 (38.6)	25 (39.7)	53 (38.1)	0.809
Moderate	90 (44.6)	29 (46.0)	61 (43.9)	
Severe	34 (16.8)	9 (14.3)	25 (18.0)	
*ARDS risk factor*—*N (%)*				
Trauma	36 (17.8)	8 (13)	28 (20)	0.28
Pneumonia	140 (69.3)	44 (70)	96 (69)	1.00
Non-pulmonary sepsis	36 (17.8)	14 (22)	22 (16)	0.37
*Oxygenation parameters*				
PaO_2_ (kPa) *{mmHg}*	11.1 [9.7, 13.5]	11.6 [10.0, 14.1]	11.0 [9.5, 12.8]	0.09
	*{83.3 [72.8, 101.3]}*	*{87 [75, 105.8]}*	*{82.5 [71.3, 96.0]}*	
FiO_2_	0.5 [0.4, 0.8]	0.5 [0.4, 0.7]	0.5 [0.4, 0.8]	0.61
P/F ratio (kPa) *{mmHg}*	23.9 [16.2, 31.0]	24.7 [18.2, 34.2]	23.5 [15.4, 30.0]	0.11
	*{179.3,[121.5, 232.5]}*	*{185.3 [136.5, 256.5]}*	*{176.3 [115.5, 225.0]}*	
SaO_2_ (%)^b^	97.4 [95.5, 98.5]	97.7 [96.0, 98.8]	97.3 [95.3, 98.2]	0.27
*Ventilation parameters*				
Tidal volume (ml/kg PBW)	7.1 [6.3, 8.4]	7.3 [6.4, 8.6]	7.1 [6.3, 8.3]	0.32
PEEP (cmH_2_O)	8.0 [5.0, 10.0]	7.0 [5.0, 8.0]	8.0 [5.0, 10.0]	0.05
Mean airway pressure (cmH_2_O)^d^	13.0 [9.0, 16.0]	13.0 [9.0, 15.0]	12.0 [10.0, 16.0]	0.57
Lactate (mmol/L)	1.2 [0.8, 1.8]	1.4 [1.0, 2.0]	1.1 [0.8, 1.6]	0.02
Vasopressor use—N (%)*	114 (56.4)	41 (65)	73 (52)	0.13
*Adjunctive therapies*^#^				
Neuromuscular blockade—N (%)	62 (30.7)	14 (22)	48 (35)	0.11
Nitric oxide—N (%)	25 (12.4)	9 (14)	16 (11)	0.75
Prone positioning—N (%)	14 (6.9)	5 (8)	9 (6)	0.94

Data are presented as median [IQR] unless otherwise stated

Values of oxygenation are presented in kPa, and the corresponding values in mmHg is presented in italics

APACHE II, Acute physiology and chronic health evaluation II; SOFA, Sequential organ failure assessment score; PaO_2_, partial pressure of arterial oxygen; FiO_2_,Fraction of inspired oxygen; P/F ratio, partial pressure of arterial oxygen to fraction of inspired oxygen ratio; SaO_2_, Saturation of arterial oxygen; PBW, predicted body weight; PEEP, Positive end-expiratory pressure

*At time of ARDS diagnosis

^#^Use within first 7 days of ARDS diagnosis

^a^12.9% of patients with missing data (17 survivors, 9 non-survivors)

^b^4.0% of patients with missing data (4 survivors, 4 non-survivors)

^c^7.4% of patients with missing data (9 survivors. 6 non-survivors)

The average time-weighted PaO_2_ during the first 7 days of ARDS was similar between non-survivors and survivors (11.3 kPa [10.2, 12.5] (84.8 mmHg [76.5, 93.8]) vs. 11.9 kPa [10.9, 12.6] (89.3 mmHg [81.8, 94.5]*)*; *p* = 0.08). In contrast, during the first 7 days after meeting ARDS criteria, the highest recorded P/F ratio was lower in non-survivors than in survivors (38.2 kPa [IQR 26.4, 44.6] (286.5 mmHg [198.0, 334.5]) vs. 41.7 kPa [35.1, 49.5] (312.8 mmHg [263.3, 371.3]); *p* = 0.004), and non-survivors also had fewer hyperoxaemia episodes (defined as a PaO_2_ > 14 kPa (105 mmHg)) (2 [0.5, 4] vs 3 [1, 6]; *p* = 0.04).

The number of patients ever exposed to excess oxygen was similar between survivors and non-survivors (54 vs. 57%; *p* = 0.79), whilst the cumulative excess oxygen exposure within the first seven days of ARDS was low and similar between survivors and non-survivors (0.1 [0.0, 0.3] vs. 0.1 [0.0, 0.2]; *p* = 0.84) (Table 2).

**Table 2:** Oxygenation parameters

Parameter	Overall N = 202	Non-Survivors N = 63	Survivors N = 139	*p*-value
Highest PaO_2_ (kPa) {mmHg}	17.40 [14.8, 21.9] *{130.5 [111.0, 164.3]}*	16.5 [14.3, 21.8] *{123.8 [107.3, 163.5]}*	18.2 [15.0, 21.8] *{136.5 [112.5, 163.5]}*	0.22
Highest FiO_2_ (%)	0.7 [0.5, 0.8]	0.7 [0.6, 0.9]	0.7 [0.5, 0.8]	0.17
Highest P/F ratio (kPa) *{mmHg}*	41.0 [32.7, 48.6] *{307.5 {245.3, 364.5]}*	38.2 [26.4, 44.6] *{286.5 [198.0, 334.5]}*	41.7 [35.1, 49.5] *{312.8 [263.3, 371.3]}*	0.004
*Average time-weighted PaO*_*2*_* (kPa) {mmHg}*				
First 24 h of ARDS	11.5 [10.4, 13.1] *{86.3 [78.0, 98.3]}*	11.4 [10.1, 12.9] *{85.5 [75.8, 96.8]}*	11.6 [10.5, 13.1] *{87.0 [78.8, 98.3]}*	0.37
First 7 days of ARDS	11.8 [10.7, 12.6] *{88.5 [80.3, 94.5]}*	11.3 [10.2, 12.5] *{84.8 [76.5, 93.8]}*	11.9 [10.9, 12.6] *{89.3 [81.8, 94.5]}*	0.08
Number of hyperoxaemia episodes (First 7 days of ARDS)	3.0 [1.0, 6.0]	2.0 [0.5, 4.0]	3.0 [1.0, 6.0]	0.04
Any hyperoxaemia episode–N (%)	166 (82)	47 (75)	119 (86)	0.09
Ever exposed to excess oxygen–N (%)	111 (55)	36 (57)	75 (54)	0.79
*Excess oxygen exposure*				
First 24 h of ARDS	0.0 [0.0, 0.2]	0.0 [0.0, 0.2]	0.0 [0.0, 0.1]	0.75
First 7 days of ARDS	0.1 [0.0, 0.2]	0.1 [0.0, 0.2]	0.1 [0.0, 0.3]	0.84

Data are presented as median [IQR] unless otherwise stated

Values of oxygenation are presented in kPa, and the corresponding values in mmHg is presented in italics

Average time-weighted PaO_2_ was obtained by calculating the value between consecutive time points prior to multiplying this value by the period of time between these points. The sum of these time-weighted values was then divided by the total time of mechanical ventilation within the timepoints of interest. Hyperoxaemia was defined as a PaO_2_ > 14 kPa (105 mmHg). Excess oxygen exposure was defined as any FiO_2_ value above an FiO_2_ > 0.5 in patients with a PaO_2_ > 10.7 kPa (80.3 mmHg), and was calculated at 24-h intervals with a cumulative value obtained

PaO_2_, partial pressure of arterial oxygen; FiO_2_, Fraction of inspired oxygen; P/F ratio, partial pressure of arterial oxygen to fraction of inspired oxygen ratio

### ICU mortality

**Fig. 1:**
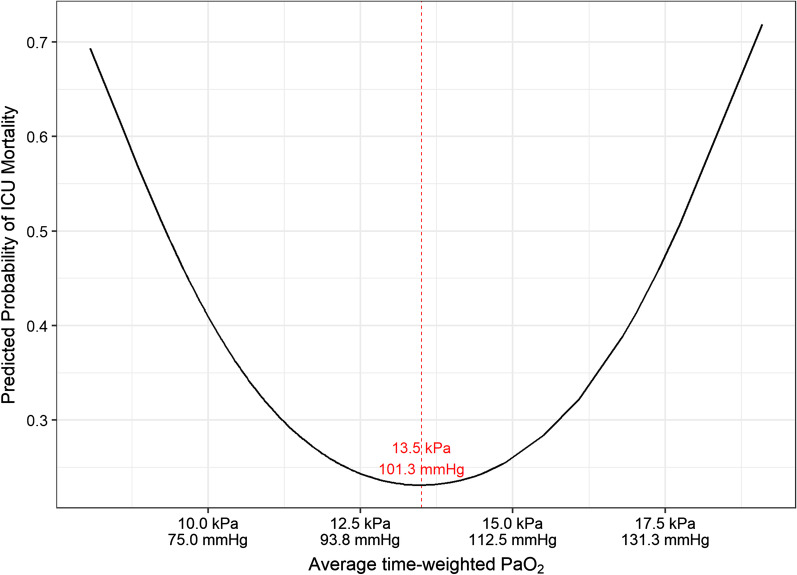
Relationship between average time-weighted PaO_2_ and ICU mortality. Predicted probability of ICU mortality by average time-weighted PaO_2_ (within the first 7 days of ARDS)

In univariable and multivariable analysis, average time-weighted PaO_2_ demonstrated a U-shaped relationship with ICU mortality. For values of average time-weighted PaO_2_ < 13.5 kPa (101.3 mmHg), increasing average time-weighted PaO_2_ is associated with a reduced mortality. In contrast, for values of average time-weighted PaO_2_ > 13.5 kPa (101.3 mmHg), when average time-weighted PaO_2_ increases this is associated with increased ICU mortality. The effect of average time-weighted PaO_2_ on ICU mortality is shown in Fig. 1.

Of the other variables that were assessed, age, non-respiratory SOFA score, and blood lactate were associated with increased ICU mortality, whilst highest recorded P/F ratio and higher baseline PEEP were associated with reduced ICU mortality (Table 3).

**Table 3:** Univariable and multivariable analysis for ICU mortality

Predictor	Univariable analysis		Multivariable analysis	
	Odds ratio [95% CI]	*p*-value	Odds ratio [95% CI]	*p*-value
Age (years)	1.05 [1.03, 1.07]	< 0.001	1.04 [1.02, 1.07]	< 0.001
Male	0.85 [0.46,1.58]	0.61		
APACHE II	1.03 [0.98, 1.09]	0.21		
Non-respiratory SOFA score	1.12 [1.03, 1.22]	0.01	1.09 [0.98, 1.22]	0.10
*Admission source*				
Emergency	1.09 [0.57, 2.17]	0.79		
Elective	0.82 [0.17, 2.94]	0.77		
Medical	0.93 [0.51, 1.7]	0.82		
Surgical	1.47 [0.79, 2.71]	0.22		
*ARDS severity*^a^				
Moderate	1.01 [0.53,1.94]	0.98		
Severe	0.76 [0.30,1.83]	0.56		
*ARDS risk factor*				
Trauma	0.58 [0.23, 1.3]	0.20		
Pneumonia	1.04 [0.55, 2.01]	0.91		
Non-pulmonary sepsis	1.52 [0.71, 3.19]	0.27		
*Baseline ventilation parameters*				
Tidal volume (ml/kg PBW)	1.08 [0.92, 1.26]	0.33		
PEEP (cmH_2_O)	0.88 [0.78, 0.99]	0.04	0.87 [0.76, 0.99]	0.05
Mean airway pressure (cmH_2_O)	0.99 [0.93, 1.02]	0.58		
Lactate (mmol/L)	1.18 [1.01, 1.39]	0.04	1.17 [0.96, 1.43]	0.12
Vasopressor use	1.68 [0.92, 3.15]	0.10		
*Adjunctive therapies*				
Neuromuscular blockade	0.54 [0.27, 1.06]	0.08		
Nitric oxide	1.28 [0.51, 3.03]	0.58		
Prone positioning	1.25 [0.37, 3.77]	0.70		
*Highest PaO*_*2*_* (kPa/mmHg)*				
Quadratic term	1.00 [1.00, 1.01]	0.04		
Linear term	0.85 [0.73, 0.99]	0.04		
Highest FiO_2_	1.01 [0.93, 1.1]	0.72		
Highest P/F ratio	0.97 [0.95, 0.99]	0.02		
*Average time-weighted PaO*_*2*_* (kPa/mmHg)*				
Quadratic term	1.07 [1.01, 1.14]	0.02	1.08 [1.01, 1.16]	0.03
Linear term	0.16 [0.03, 0.67]	0.01	0.12 [0.02, 0.66]	0.02

Average time-weighted PaO_2_ was obtained by calculating the value between consecutive time points prior to multiplying this value by the period of time between these points. The sum of these time-weighted values was then divided by the total time of mechanical ventilation (up to 7 days from onset of ARDS) within the timepoints of interest

APACHE II, Acute physiology and chronic health evaluation II; SOFA, Sequential organ failure assessment score; PaO_2_, partial pressure of arterial oxygen; FiO_2_, Fraction of inspired oxygen; P/F ratio, partial pressure of arterial oxygen to fraction of inspired oxygen ratio; PBW, predicted body weight; PEEP, Positive end-expiratory pressure

^a^The reference category for the comparison was Mild

After adjusting for age, lactate, non-respiratory SOFA score and baseline PEEP, average time-weighted PaO_2_ within the first 7 days of ARDS continued to demonstrate a U-shaped relationship with ICU mortality, similar to that reported in the univariable analysis (quadratic term OR 1.08 [1.01–1.16]; *p* = 0.03 and linear term OR 0.12 [0.02–0.66]; *p* = 0.02). Of the other co-variables, age was associated with increased ICU mortality whilst PEEP was associated with reduced ICU mortality (Table 3).

### Hospital mortality

**Fig. 2:**
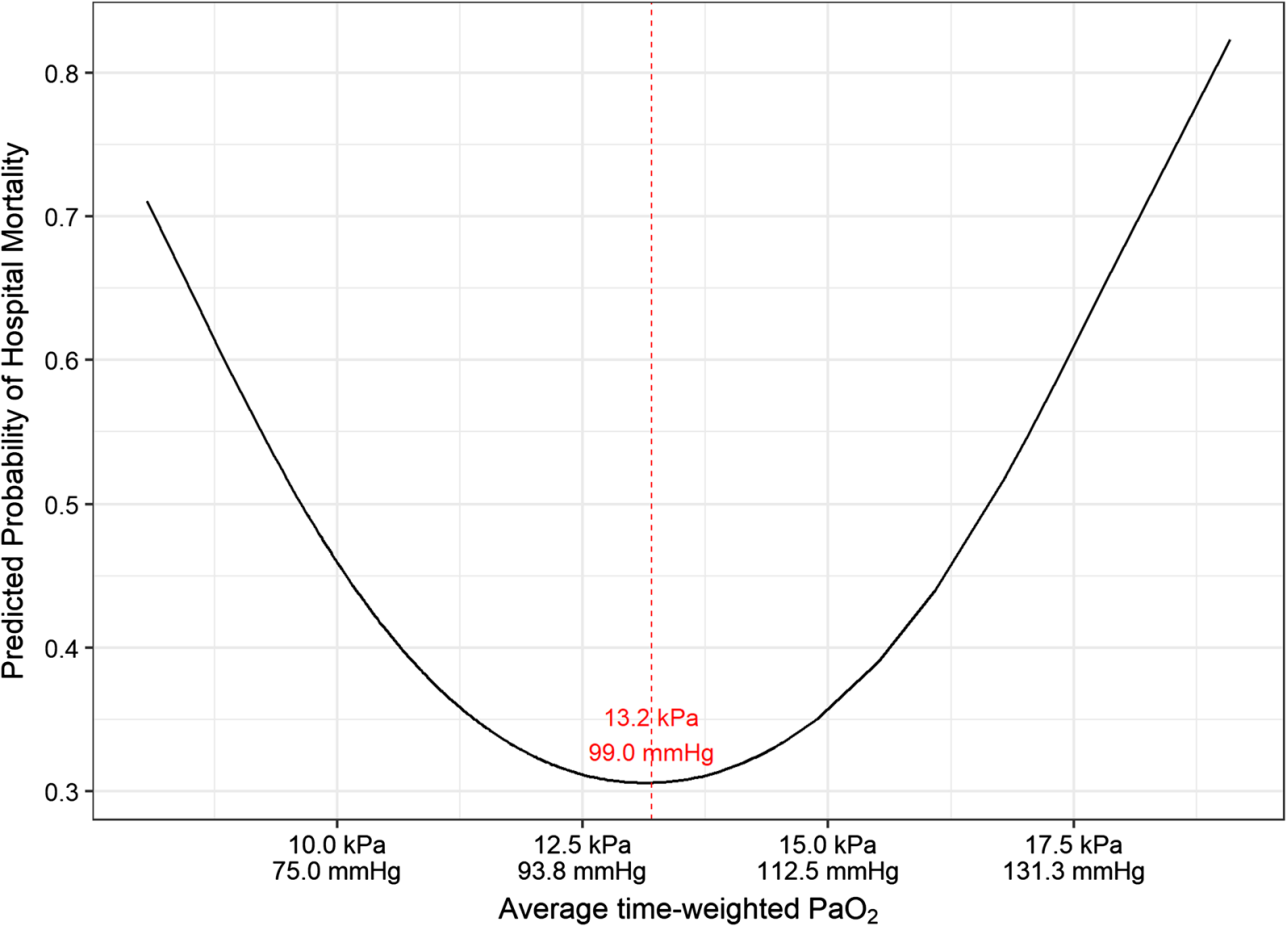
Relationship between average time-weighted PaO_2_ and hospital mortality. Predicted probability of hospital mortality by average time-weighted PaO_2_ (within the first 7 days of ARDS)

In univariate and multivariable analysis, average time-weighted PaO_2_ demonstrated a U-shaped relationship with hospital mortality (Fig. 2). When average time-weighted PaO_2_ was < 13.2 kPa (99.0 mmHg), increasing average time-weighted PaO_2_ was associated with reduced mortality. In contrast, average time-weighted PaO_2_ beyond 13.2 kPa (99.0 mmHg) was associated with increased mortality.

After adjusting for age, lactate, non-respiratory SOFA score and baseline PEEP in multivariable analysis, average time-weighted PaO_2_ within the first 7 days of ARDS continued to have a significant association with hospital mortality (quadratic term 1.09 [1.02–1.18]; *p* = 0.02 and linear term 0.11 [0.01–0.63]; *p* = 0.02). The pattern of the relationship is similar to that presented in Fig. 2. Of the other co-variables, age was associated with increased ICU mortality whilst PEEP was associated with reduced hospital mortality (Table 4).

**Table 4:** Univariable and multivariable analysis for hospital mortality

Predictor	Univariable analysis		Multivariable analysis	
	Odds ratio [95% CI]	*p*-value	Odds ratio [95% CI]	*p*-value
Age (years)	1.05 [1.03, 1.08]	< 0.001	1.05 [1.03, 1.08]	< 0.001
Male	1.02 [0.57, 1.85]	0.95		
APACHE II	1.04 [0.99, 1.09]	0.12		
Non-respiratory SOFA score	1.12 [1.03, 1.22]	0.007	1.11 [1.00, 1.23]	0.05
*Admission source*				
Emergency	0.85 [0.46, 1.61]	0.62		
Elective	1.41 [0.39, 4.84]	0.58		
Medical	0.96 [0.54, 1.70]	0.89		
Surgical	1.48 [0.82, 2.68]	0.19		
*ARDS severity*^a^				
Moderate	0.79 [0.42, 1.47]	0.46		
Severe	0.57 [0.23, 1.32]	0.20		
*ARDS risk factor*				
Trauma	0.58 [0.25, 1.25]	0.18		
Pneumonia	1.26 [0.68, 2.39]	0.46		
Non-pulmonary sepsis	1.62 [0.78, 3.37]	0.19		
*Baseline ventilation parameters*				
Tidal volume (ml/kg PBW)	1.03 [0.88, 1.20]	0.71		
PEEP (cmH_2_O)	0.87 [0.77, 0.97]	0.01	0.86 [0.75, 0.98]	0.03
Mean airway pressure (cmH_2_O)	0.95 [0.89, 1.00]	0.15		
Lactate (mmol/L)	1.17 [1.00, 1.37]	0.05	1.13 [0.93, 1.39]	0.21
Vasopressor use	1.70 [0.95, 3.08]	0.08		
*Adjunctive therapies*				
Neuromuscular blockade	0.52 [0.27, 0.98]	0.05		
Nitric oxide	1.12 [0.46, 2.61]	0.79		
Prone positioning	0.92 [0.27, 2.76]	0.88		
*Highest PaO*_*2*_* (kPa/mmHg)*				
Quadratic term	1.00 [1.00, 1.00]	0.09		
Linear term	0.88 [0.76, 1.01]	0.09		
Highest FiO2	1.00 [0.91, 1.09]	0.96		
Highest P/F ratio	0.98 [0.96, 1.00]	0.10		
*Average time-weighted PaO*_*2*_* (kPa/mmHg)*				
Quadratic term	1.07 [1.01, 1.14]	0.03	1.09 [1.02, 1.18]	0.02
Linear term	0.17 [0.04, 0.73	0.02	0.11 [0.01, 0.63]	0.02

Average time-weighted PaO_2_ was obtained by calculating the value between consecutive time points prior to multiplying this value by the period of time between these points. The sum of these time-weighted values was then divided by the total time of mechanical ventilation (up to 7 days from onset of ARDS) within the timepoints of interest

APACHE II, Acute physiology and chronic health evaluation II; SOFA, Sequential organ failure assessment score; PaO_2_, partial pressure of arterial oxygen; FiO_2_, Fraction of inspired oxygen; P/F ratio, partial pressure of arterial oxygen to fraction of inspired oxygen ratio; PBW, predicted body weight; PEEP, Positive end-expiratory pressure

^a^The reference category for the comparison was Mild

### Effect of ARDS severity

To evaluate whether there was a difference in effect based on ARDS severity at diagnosis, separate multivariable analyses were performed for patients with mild (n = 78), moderate (n = 90) and severe ARDS (n = 34). In patients with moderate ARDS, average time-weighted PaO_2_ was significantly associated with ICU mortality (quadratic term 1.21 [1.08–1.43]; *p* < 0.01 and linear term 0.01 [0.00–0.14]) (Additional file 1: Table S1) and with hospital mortality (quadratic term 1.16 [1.04–1.35]; *p* = 0.02 and linear term 0.03 [0.00–0.38]; *p* = 0.02) (Additional file 1: Table S2), There was no association between average time-weighted PaO_2_ and ICU or hospital mortality in patients with either mild or severe ARDS.

### Duration of mechanical ventilation

There was no association between average time-weighted PaO_2_ over 7 days and duration of mechanical ventilation (− 0.05 [− 0.12, 0.02]; *p* = 0.19) (Additional file 1: Table S3). In an adjusted analysis, highest recorded P/F ratio was associated with an increased duration of mechanical ventilation (0.01 [0.00, 0.01]; *p* = 0.04). Use of neuromuscular blockade during the first seven days of ARDS was also associated with an increased duration of mechanical ventilation (0.44 [0.19–0.70]; *p* = 0.001), whilst increasing tidal volume (− 0.09 [− 0.15, − 0.03]; *p* = 0.002) and lactate (− 0.08 [− 0.14, -0.02]; *p* = 0.01) were associated with a reduced duration of mechanical ventilation (Additional file 1: Table S4).

Patients who survived ICU had a longer duration of mechanical ventilation compared to non-survivors (10 [6, 17] days vs. 7 [4, 12]; *p* = 0.001). In an analysis restricted to patients who survived ICU, there was no association between average time-weighted PaO_2_ over 7 days and duration of mechanical ventilation (− 0.06 [− 0.15, 0.03]; *p* = 0.18) (Additional file 1: Table S5). In multivariable analysis, the use of neuromuscular blockade was associated with an increase (0.52 [0.22–0.82]; *p* = 0.001), and tidal volume a reduction (− 0.09 [− 0.16, − 0.01]; *p* = 0.03), in duration of mechanical ventilation in survivors (Additional file 1: Table S6).

### Mediation analysis

In an effort to establish whether there was a causative effect between hyperoxaemia exposure and outcome, mediation analysis was performed using both blood lactate [23] and baseline tidal volume [4] as mediators that could affect outcome. In both cases no significant effect was identified between either high lactate (*p* = 0.33) or baseline tidal volume (*p* = 0.08) as mediators that affects outcome of patients exposed to hyperoxaemia in the first 24 h of ARDS in a model with baseline FiO_2_ and PEEP as confounding variables. Therefore, the three conditions for mediation analysis, defined in the methods section, are not satisfied.

## Discussion

In prospectively identified patients with ARDS, the predicted probability of both ICU and hospital mortality was lowest when the average time-weighted PaO_2_ was between 12.5–14 kPa (93.8–105.0 mmHg). This suggests that clinicians targeting a PaO_2_ out-with this range may increase the risk of mortality in their patients with ARDS, and this data is novel in providing a clearer understanding of the risks of different oxygenation targets in patients with ARDS.

These findings expand upon the findings from previous studies, and provide further insight about optimal oxygen targets in patients with ARDS [2]. In a clinical trial of patients with ARDS, a liberal oxygen strategy (targeting a PaO_2_ 12–14 kPa (90.0–105.0 mmHg)) was associated with increased survival compared with a conservative oxygen strategy (targeting a PaO_2_ 7.3–9.3 kPa (55.0–70.0 mmHg)) [15]. In contrast, although not limited to patients with ARDS, the HOT-ICU study evaluated patients with acute hypoxaemic respiratory failure and did not identify any difference in outcome when patients were managed with either a target PaO_2_ of either 8 kPa (60.0 mmHg) or 12 kPa (90.0 mmHg) [14]. Interestingly, the safest threshold of average time-weighted PaO_2_ within the first 7 days of ARDS for patients in this analysis was 12.5–14 kPa (93.8–105.0 mmHg), which was a similar target to that which showed benefit in a previous study of patients with ARDS [15]. When patients were grouped by ARDS severity, the effect of average time-weighted PaO_2_ was only observed in patients with moderate ARDS. Although this may be driven by the slightly higher number of patients with moderate ARDS, it is possible that there is a difference in effect of oxygen exposure based on illness severity.

The concept of a U-shaped relationship between oxygen targets and mortality has been previously demonstrated in a broad cohort of critically ill patients. In a retrospective analysis of two large patient datasets of patients admitted to ICUs, it was demonstrated that hospital mortality was lowest in patients when their median SpO_2_ was 94–98% [24]. Titrating oxygen to SpO_2_ targets is likely to be more achievable in resource-limited environments and therefore a study of oxygen titration to SpO_2_ in patients with ARDS may be warranted in future. Such studies may wish to prioritise including patients with moderate ARDS given the findings presented in this manuscript.

Whilst oxygen targets are predominantly based on pulse oximetry or PaO_2_ the variable that is most likely to be adjusted to achieve these targets is FiO_2_. It is difficult to extrapolate the benefits or harms from one oxygenation target without considering the potential harms from higher FiO_2_. Indeed, it is plausible that patients at both extremes of arterial oxygenation were each exposed to high FiO_2_. Exposure to an FiO_2_ set at 1.0 has been shown to be associated with increased mortality in critically ill patients with septic shock [5], and therefore it is possible that the harm demonstrated in this analysis (of an average time-weighted PaO_2_ within the first 7 days of ARDS out-with 12.5–14 kPa (93.8–105.0 mmHg)) is at least in part mediated by exposure to high FiO_2_.

In an effort to better understand whether there was a causal relationship between hyperoxaemia exposure (PaO_2_ > 14 kPa (105 mmHg)) and clinical outcome, mediation analysis was performed. Hyperoxaemia within the first 24 h of ARDS was selected as a potentially modifiable exposure that occurred early in the disease course, and as early hyperoxaemia had previously been demonstrated to be associated with increased mortality in patients without ARDS [19]. Potential mediators that could have affected this exposure include baseline tidal volume [4] and high lactate [23]. Unfortunately, the three conditions for mediation analysis were not met, which may reflect the choice of mediator. This study is limited in mediator assessment because of the data variables collected, and therefore future studies may wish to evaluate whether there is a mediation effect of alternative variables. For example, the effect of different oxygen titration strategies in patients with sepsis remains uncertain [5, 25], and therefore it is plausible that differences in the host response may modify the risks from hyperoxaemia (e.g. the effect of hyperoxaemia in patients with neutropenia may differ from that in patients with neutrophilia). This points to the need for improved understanding of the mechanisms of potential harm from hyperoxia and hyperoxaemia.

This study has several strengths. Patients were prospectively identified according to the Berlin criteria [17]. All patients had established ARDS and were receiving invasive mechanical ventilation, which contrasts with previous studies in a general ICU population [11], and therefore may better inform the bedside clinician about the association between arterial oxygenation targets and outcomes in patients with ARDS. In keeping with all observational studies, these results are limited by unmeasured variables that could have a confounding effect, both on the effect of oxygen exposure but also on the patient course during their illness. This includes patient co-morbidities, the duration of mechanical ventilation prior to ARDS diagnosis, the use of oxygen during tracheal intubation, and the frequency of respiratory physiotherapy or recruitment maneuvers, each of which were not included in this evaluation. Whilst PEEP was controlled for in multivariable analysis, a more precise understanding of the effects of arterial oxygenation targets may be obtained in a study where PEEP is similar between groups. Future studies may consider evaluating the relationship between oxygen exposure and ARDS sub-phenotype [26] as it is plausible that there may be a difference in effect in patients with either a hyper- or hypo-inflammatory sub-phenotype. Finally, the findings in this study may be limited by the number of patients analysed. As this was designed as an exploratory analysis using a convenience sample, there was no sample size calculation performed. Therefore, it is possible that the study does not include a sufficient number of patients to identify a difference in hyperoxaemia exposure that has a significant impact on survival in patients with ARDS. Furthermore, in the absence of a sample size calculation, it is difficult to quantify the bias of the population sample studied. Therefore, although the results from this study are in keeping with those from recent randomized controlled trials [15], the use of a convenance sample means there is greater uncertainty regarding the applicability of the results of this study to a wider population of patients. This emphasizes that the results from this study should be considered hypothesis-generating, and support the need for appropriately powered clinical trials investigating the optimal oxygenation targets in patients with ARDS.

## Conclusion

Despite existing evidence, there remains a need to better define the optimal oxygenation targets in ARDS, and how to safely titrate oxygen to achieve these but without exposing patients to potentially injurious hyperoxia. Furthermore, there remains a need to define hyperoxaemia, with a pragmatic definition that can be evaluated by the bedside clinician likely to be of most practical benefit. In patients with ARDS, the predicted probability of both ICU and hospital mortality was lowest when the average time-weighted PaO_2_ was between 12.5 and 14 kPa (93.8–105.0 mmHg), suggesting this is a reasonable oxygenation target for clinicians to aim for. A recently completed clinical trial evaluating different oxygenation targets in patients with systemic inflammatory response syndrome (NCT02321072) will add to existing data in patients at risk for ARDS and will help to inform clinicians about the risks to their patients of different oxygenation targets.

## Supplementary Information

**Additional file 1**. Contains details regarding patient exclusions and additional tables with results that support the data presented in the manuscript.

## Data Availability

The dataset used to generate this manuscript may be made available from the corresponding author on reasonable request.

## Abbreviations

ARDS: Acute respiratory distress syndrome
ICU: Intensive care unit
P/F ratio: Ratio of partial pressure of arterial blood oxygen content to inspired fraction of oxygen
PEEP: Positive end-expiratory pressure
APACHE II: Acute physiology and chronic health evaluation II
SOFA: Sequential organ failure assessment
PBW: Predicted body weight
FiO_2_: Fraction of inspired oxygen
PaO_2_: Partial pressure of arterial oxygen
SaO_2_: Arterial oxyhemoglobin saturation
SpO_2_: Arterial oxyhemoglobin saturation measured by pulse oximetry
MAP: Mean airway pressure
IQR: Interquartile range
CI: Confidence interval
