# Supplementary material for: Hyperoxaemia and hypoxaemia are associated with harm in patients with ARDS
Source: BMC Pulm Med. 2021 Sep 8;21:285. doi: 10.1186/s12890-021-01648-7 (PMC8424163; doi:10.1186/s12890-021-01648-7)
Supplement: Supplementary file 1 — Additional file 1. Contains details regarding patient exclusions and additional tables with results that support the data presented in the manuscript. [file 12890_2021_1648_MOESM1_ESM.docx]

**Hypoxaemia and hyperoxaemia are associated with harm in patients with ARDS**

**Additional file 1**

**EXCLUSION CRITERIA**

Of the 222 patients identified for inclusion, 20 were excluded from the final analysis following review by trained medical staff:

Less than 12 hours of available data – 5 patients

Not meeting ARDS criteria – 13 patients

Duplicate patient identified – 2 patients

**RESULTS AND TABLES**

Table S1. Multivariable analysis for ICU mortality by ARDS severity

|  | **Mild ARDS** | | **Moderate ARDS** | | **Severe ARDS** | |
| --- | --- | --- | --- | --- | --- | --- |
| **Predictor** | **Odds Ratio**  [95% CI] | **p-value** | **Odds Ratio**  [95% CI] | **p-value** | **Odds Ratio**  [95% CI] | **p-value** |
| **Age** (years) | 1.04 [1.00, 1.09] | 0.05 | 1.06 [1.02, 1.10] | <0.01 | 1.07 [1.00, 1.19] | 0.11 |
| **Non-respiratory SOFA score** | 1.08 [0.90, 1.31] | 0.39 | 1.17 [1.00, 1.38] | 0.06 | 1.08 [0.75, 1.58] | 0.67 |
| **PEEP** (cm H_2_O) | 0.83 [0.59, 1.14] | 0.28 | 0.92 [0.73, 1.15] | 0.45 | 0.80 [0.52, 1.12] | 0.24 |
| **Lactate** (mmol/L) | 1.53 [1.05, 2.49] | 0.05 | 1.03 [0.73, 1.44] | 0.88 | 1.00 [0.57, 1.75] | 0.99 |
| **Average time-weighted PaO_2_** (kPa / mmHg)  Quadratic term  Linear term | 1.00 [0.82, 1.13]  0.54 [0.02, 76.87] | 0.95  0.74 | 1.21 [1.08, 1.43]  0.01 [0.00, 0.14] | <0.01  <0.01 | 1.57 [1.03, 2.96]  0.00 [0.00, 0.53] | 0.09  0.09 |

**Table S2. Multivariable analysis for Hospital mortality by ARDS severity**

|  | **Mild ARDS** | | **Moderate ARDS** | | **Severe ARDS** | |
| --- | --- | --- | --- | --- | --- | --- |
| **Predictor** | **Odds Ratio**  [95% CI] | **p-value** | **Odds Ratio**  [95% CI] | **p-value** | **Odds Ratio**  [95% CI] | **p-value** |
| **Age** (years) | 1.04 [1.01, 1.08] | 0.03 | 1.07 [1.03, 1.11] | <0.01 | 1.10 [1.02, 1.24] | 0.04 |
| **Non-respiratory SOFA score** | 1.04 [0.88, 1.24] | 0.65 | 1.23 [1.05, 1.46] | 0.01 | 1.16 [0.82, 1.71] | 0.42 |
| **PEEP** (cm H_2_O) | 0.77 [0.55, 1.03] | 0.10 | 0.93 [0.74, 1.17] | 0.53 | 0.77 [0.49, 1.09] | 0.19 |
| **Lactate** (mmol/L) | 1.56 [1.06, 2.62] | 0.04 | 1.03 [0.73, 1.46] | 0.89 | 0.87 [0.48, 1.50] | 0.62 |
| **Average time-weighted PaO_2_** (kPa / mmHg)  Quadratic term  Linear term | 1.06 [0.95, 1.20]  0.13 [0.01, 2.60] | 0.27  0.18 | 1.16 [1.04, 1.35]  0.03 [0.00, 0.38] | 0.02  0.02 | 1.55 [1.01, 3.01]  0.00 [0.00, 0.79] | 0.10  0.11 |

**Table S3. Univariable analysis for duration of mechanical ventilation (log scale)**

| **Predictor** | **Estimate**  **(95% CI)** | **p-value** |
| --- | --- | --- |
| Age (years) | -0.01 (-0.02, -0.002) | 0.01 |
| Male | 0.06 (-0.19, 0.30) | 0.66 |
| APACHE II | -0.01 (-0.03, 0.01) | 0.48 |
| Non-respiratory SOFA score | -0.01 (-0.04, 0.03) | 0.71 |
| **Admission source**  Emergency  Elective  Medical  Surgical | 0.10 (-0.17, 0.36)  0.28 (-0.24, 0.81)  0.04 (-0.20, 0.28)  -0.14 (-0.39, 0.11) | 0.47  0.29  0.73  0.26 |
| **ARDS risk factor**  Trauma  Pneumonia  Non-pulmonary sepsis | 0.19 (-0.13, 0.50)  0.01 (-0.26, 0.27)  -0.29 (-0.60, 0.02) | 0.24  0.97  0.07 |
| Highest PaO_2_ (kPa / mmHg) | 0.01 (-0.01, 0.02) | 0.48 |
| Highest FiO_2_ | -0.002 (-0.03, 0.03) | 0.91 |
| Highest P/F ratio (kPa / mmHg) | 0.01 (0.0003, 0.01) | 0.04 |
| Average time-weighted PaO_2_  (within the first 7 days of ARDS) (kPa / mmHg) | -0.05 (-0.12, 0.02) | 0.19 |
| **Baseline ventilation parameters**  Tidal volume (ml/kg PBW)  PEEP (cmH_2_O)  Mean airway pressure (cmH_2_O) | -0.11 (-0.17, -0.05)  0.06 (0.02, 0.10)  0.02 (0.002, 0.03) | 0.001  0.006  0.03 |
| Lactate (mmol/L) | -0.07 (-0.13, 0.0004) | 0.05 |
| Vasopressor use | 0.03 (-0.21, 0.28) | 0.78 |
| **Adjunctive therapies**  Neuromuscular blockade  Nitric oxide  Prone positioning | 0.56 (0.31, 0.81)  0.12 (-0.25, 0.48)  0.34 (-0.13, 0.81) | <0.001  0.52  0.15 |

Values of the estimate are values of the estimated beta coefficient of predictors, and therefore positive coefficient values mean that for a unit increase in the predictor, the outcome is increasing, and for negative coefficient values the outcome is decreasing. For example, a unit increase in PEEP (beta=0.06), would result in (exp(beta)-1)*100 = 6% increase in duration of mechanical ventilation.

Average time-weighted PaO_2_ was obtained by calculating the value between consecutive time points prior to multiplying this value by the period of time between these points. The sum of these time-weighted values was then divided by the total time of mechanical ventilation within the timepoints of interest.

**Table S4. Multivariable analysis for duration of ventilation (log scale)**

| **Predictor** | **Estimate**  **(95% CI)** | **p-value** |
| --- | --- | --- |
| Highest P/F ratio (kPa / mmHg) | 0.01 [0.00, 0.01] | 0.04 |
| Age | 0.00 (-0.01, 0.00) | 0.14 |
| Tidal volume (ml/kg PBW) | -0.09 (-0.15, -0.03) | 0.002 |
| Lactate (mmol/L) | -0.08 (-0.14, -0.02) | 0.01 |
| Neuromuscular blockade | 0.44 (0.19, 0.70) | 0.001 |

Values of the estimate are values of the estimated beta coefficient of predictors, and therefore positive coefficient values mean that for a unit increase in the predictor, the outcome is increasing, and for negative coefficient values the outcome is decreasing, both in log scale.

**Table S5. Univariate analysis for duration of ventilation in ICU survivors (log scale)**

| **Predictor** | **Estimate (95% CI)** | **p-value** |
| --- | --- | --- |
| Age (years) | -0.01 (-0.02, -0.002) | 0.02 |
| Male | 0.10 (-0.20, 0.40) | 0.50 |
| APACHE II | -0.004 (-0.03, 0.02) | 0.76 |
| Non-respiratory SOFA score | 0.03 (-0.02, 0.07) | 0.20 |
| **Admission source**  Emergency  Elective  Medical  Surgical | 0.21 (-0.11, 0.52)  0.31 (-0.30, 0.92)  0.06 (-0.22, 0.35)  -0.11 (-0.41, 0.20) | 0.19  0.32  0.68  0.48 |
| **ARDS risk factor**  Trauma  Pneumonia  Non-pulmonary sepsis | 0.10 (-0.26, 0.45)  -0.07 (-0.38, 0.24)  -0.27 (-0.68, 0.12) | 0.58  0.67  0.17 |
| Highest PaO_2_ (kPa / mmHg) | 0.01 (-0.01, 0.03) | 0.23 |
| Highest FiO_2_ | 0.0004(-0.04, 0.04) | 0.99 |
| Highest P/F ratio (kPa / mmHg) | 0.01 (-0.001, 0.01) | 0.10 |
| Average time-weighted PaO_2_  (within first 7 days of ARDS) (kPa / mmHg) | -0.06 (-0.15, 0.03) | 0.18 |
| **Baseline ventilation parameters**  Tidal volume (ml/kg PBW)  PEEP (cmH_2_O)  Mean airway pressure (cmH_2_O) | -0.11 (-0.19, -0.02)  0.06 (0.01 , 0.11)  0.02 (0.004, 0.03) | 0.01  0.01  0.02 |
| Lactate (mmol/L) | -0.03 (-0.13, 0.06) | 0.48 |
| Vasopressor use | 0.15 (-0.13, 0.43) | 0.30 |
| **Adjunctive therapies**  Neuromuscular blockade  Nitric oxide  Prone position | 0.62 (0.34, 0.90)  0.46 (0.03, 0.90)  0.59 (0.02, 1.16) | <0.001  0.04  0.04 |

Values of the estimate are values of the estimated beta coefficient of predictors, and therefore positive coefficient values mean that for a unit increase in the predictor, the outcome is increasing, and for negative coefficient values the outcome is decreasing, both in log scale.

Average time-weighted PaO_2_ was obtained by calculating the value between consecutive time points prior to multiplying this value by the period of time between these points. The sum of these time-weighted values was then divided by the total time of mechanical ventilation within the timepoints of interest.

**Table S6. Multivariable analysis for duration of ventilation in ICU survivors (log scale)**

| **Predictor** | **Estimate**  **(95% CI)** | **p-value** |
| --- | --- | --- |
| Highest P/F ratio (kPa / mmHg) | 0.00 [0.00, 0.01] | 0.12 |
| Age | -0.01 (-0.01, 0.00) | 0.14 |
| PEEP | 0.02 (-0.03, 0.07) | 0.42 |
| Tidal volume | -0.09 (-0.16, -0.01) | 0.03 |
| Lactate | -0.07 (-0.16, 0.02) | 0.12 |
| Neuromuscular blockade | 0.52 (0.22, 0.82) | 0.001 |

Values of the estimate are values of the estimated beta coefficient of predictors, and therefore positive coefficient values mean that for a unit increase in the predictor, the outcome is increasing, and for negative coefficient values the outcome is decreasing, both in log scale.
